# Can up-skilling non-physician clinicians (NPCs) make a difference to practice and help towards reductions in maternal and neonatal mortality, in Malawi? The ETATMBA Project

**DOI:** 10.1186/1472-6963-14-S2-O36

**Published:** 2014-07-07

**Authors:** David Ellard

**Affiliations:** 1 Warwick Clinical Trials Unit, The University of Warwick, Coventry, UK

## Background

The ETATMBA project in northern and central Malawi is providing advanced clinical and leadership training to 50 non-physician clinicians (NPCs) who provide emergency obstetric and new-born care (EmONC). Here we report the process evaluation of the training. The aim of the project is to try to address the high levels of maternal and neonatal mortality.

## Materials and methods

We interviewed ETATMBA trainees, based in seven districts of northern and central Malawi, District health officers, cascadees and two visiting UK Obstetricians. Trainee interviews were at three points in time (early, mid and late in the project). Topics explored included perceptions of the training, support, content, implementation in their workplace and challenges and successes.

## Results

46/54 recruited trainees were still on the course. There was substantial variation in the rates of maternal and neonatal deaths between Districts at baseline. Attendance was high and all trainees spent time working alongside an obstetrician. In early interviews trainees recalled course content unprompted indicating the training had been received. Colleagues and District Medical/nursing officers reported cascading of knowledge and initial changes in practice indicating early implementation. Asking for actual cases, we found they had implemented new knowledge and skills where the mothers and babies lives were saved. Leadership training enabled them to confidently change their own practice and initiate change in their health facility.

## Conclusions

Task shifting in countries like Malawi is, at present necessary, for the provision of health care. NCPs provide and are likely to continue to provide the majority of healthcare for low resource countries in much of sub-Saharan Africa. The results of this suggest that up-skilling this cadre with new knowledge and skills can make a difference to practice and may be helping to save lives.

